# Hahnemann’s Chronic Miasms

**Published:** 1883-06

**Authors:** P. P. Wells

**Affiliations:** Brooklyn


### HAHNEMANN'S CHRONIC MIASMS.

P. P. WELLS, M. D., BROOKLYN.

If these sources of chronic diseases are remembered in the teachings, literature, or practice of modern Homœopathy, it is oftener than otherwise that a sneer or an attempt at unseemly ridicule may be passed on the first of this series, *psora*, while the second, being too often too apparent to the senses of even the most stolid observer, is allowed to pass in silence, and the third, less obtrusive to the senses, may be safely said to have been permitted to fall into forgetfulness. *Sycosis*, as a basic cause of chronic diseases, can hardly be supposed to have place in the thoughts of the average modern Homœopathist, especially of those who have most to say of Hahnemann's "fallacies" and "errors." The first natural inquiry, when one thinks of this neglect, is—Are those who thus sneer at and neglect these teachings of the great founder of our school of practice more successful in their endeavors to cure chronic diseases than was Hahnemann, or than have been those who have accepted them and made them the basis of their prescriptions for these so commonly fatal maladies? The conviction that these gentlemen have little or no success in this part of their practice is the ready answer that intrudes itself upon us. Indeed, it could hardly have been otherwise in any attempt to cure these maladies by homœopathic means and methods, the fundamental principles of the homœopathic philosophy of these diseases being so entirely discarded or ignored. As to the discarded *psora*, Autenrieth—no homœopath, but a *professor of pathology* in the Tübingen school—went even further than

Hahnemann in his assertion that external repelled eruptions were transferred to internal organs and surfaces, and that *he had seen them there* in their original forms many times, though he protected himself from charge of heresy, from his fellows of the old school, by the assurance to them that this fact had nothing whatever to do with the genesis of chronic disease taught by the founder of Homœopathy, and added that "this, like all else that had come from that source, was mere empty air." Notwithstanding this hedging assurance of the Professor, we think most intelligent minds, after acquaintance with Hahnemann's teachings of psora as a source of chronic disease, will at once agree that Autenrieth's discovery goes far in confirmation of those teachings. This discovery of the old-school Professor demonstrates, or he is mistaken, the actual presence in internal organs and on internal surfaces of the very translated eruptions which Hahnemann says are causes of so many of the important diseases with which we have to contend. Those of our school who have been most successful healers of chronic diseases have accepted this view of the origin of a large class of those maladies which they have successfully treated, and the best of these were ready to ascribe their known successes to a recognition of this genesis, though the ignorant and the silly were at the same time so free with poor wit and mistimed sneers at the expense of this fundamental teaching of the master, and though others who had only partially come to a knowledge of homœopathic truth were at the same time seemingly nervously careful to have it understood they "were not weighted down" with this or other elements of homœopathic philosophy.

The third of Hahnemann's chronic miasms, *sycosis*, has been less considered and less opposed and perhaps less understood than psora. It was considered by Bœnninghausen, probably the most successful prescriber for chronic diseases the world has known, to be but little, if any, less important than the first. This great master had studied this miasm in its origin and effects on the human organism as no other man has, and the result was he cured its ravages in the organism as no other man has. His studies of the materia medica in its relations to this miasm and its effects were most profound and exhaustive.* He recognized the fact that the most careful "scrutiny of page after page of symptoms" by the "experienced

* *Vide Am. Hom. Review*, vol. III, p. 241, *et seq.*

practitioner" will "not be able in all cases to make the most exactly fitting choice of a remedy" in absence of a just view of the *anamnesis* of the case in hand. This is often, as he regarded it, indispensable to a right selection of the curative agent.

The following case beautifully and perfectly illustrates this fact:

The writer was called to a consultation in the case of C. S., aged five months, June 15th, 1859. The child was large, plump in form; indeed, as to figure might be taken as a model. She was perfectly healthy at birth, as were her parents then and before. Her first complaint was developed immediately after her *vaccination*. This showed itself in the form of eczema in folds of her fat limbs and neck. These were all red and raw, oozing a colorless, thick, slightly sticky and slightly offensive fluid. This eruption was followed by an attack of croup after two or three months, and this by "Miller's asthma" immediately after, the croup seeming to pass into this last, sometimes so troublesome a malady. The parents, having, a year or two before, lost a little boy by this disease, became alarmed, and I was consequently called in consultation on the case. The spasmodic disease was soon controlled and there remained visible only the eczema. But there was much more which was not visible, as was shown the first time she took a cold, to which she seemed more than commonly inclined. She had a return of the croup, and this passed into Miller's asthma, as before, showing she had not been cured radically. As before, the attack was apparently overcome and the child was well again, except her eczema. This, in the attack of croup and asthma, became dry, and the oozing only returned when the spasmodic affection was relieved. The third attack of this kind followed, again from cold, and the child now became my patient. Notwithstanding the best prescriptions of remedies and hygiene I could make, the child would take cold and repeat the experiences above mentioned till she was near two years old, when it was suggested that change of air, scene, and circumstances might be of service in healing the child of this chronic disposition to taking cold. The suggestion was accepted, and she was taken to Newburgh, N. Y., and placed under the care of my friend, the late Dr. Dunham. She took her cold there and went through her former troubles, as she had at home. Dr. D. treated her spasms with Chlorine water * successfully, and she returned to her

* *Vide Am. Hom. Review*, vol. III, p. 370.

city home at the close of the summer, as it was hoped, cured. It was not so. She soon took cold, had croup and asthma as before. The spasms were relieved by Chlorine water, and were seemingly cured, but the attacks were repeated at intervals, and not less severely, till she had grown to the age when she ran about the nursery on her feet. One day, when I called at the house, the mother said, "Doctor, what makes Lottie walk so?" She put the child on the floor, and as she walked she limped when she stepped on her right foot. She complained of pain in the hip-joint if the head of the femur were pressed into the socket or rotated, or if pressure were made on the great trochanter. The child was stripped, and the buttock of the affected side was flattened very perceptibly by atrophy of the great gluteal muscles—there was no doubt of having serious disease of the hip-joint to deal with. This was prescribed for as well as I could in the still imperfect knowledge of the case—for it was imperfectly understood, though it had been so long under my care. The prescription was hardly better than a failure. Now there was one fact in the case which, as it turned out, had received less attention from both Dr. Dunham and myself than it should. This was a thin, green, closely adherent scab on the right temple. The mother was told to have this removed at our next morning visit. This was done, and the key to the whole case was disclosed by a nipple-like *wart*, something more than an eighth of an inch in length, oozing the same sticky fluid as the eczema had been all this time discharging. This *oozing wart* was at once recognized as the representative of the original cause of all the troubles the poor child had endured. With this view a new study of the case was made, and the remedy found which had all the symptoms of the case, *including this oozing wart*. A powder in which were a few pellets of that remedy was dissolved in half a goblet of water, and of this a teaspoonful was given every six hours. The cure of the case was so prompt and perfect, including the hip disease and the eczema, that no second powder was required for its completion.

For a proper understanding of this case, Bœnninghausen and Wolf's observations of the vaccine disease should be remembered; that each, after a forty years' observation, had come to the same conclusion—that the vaccine virus was the concrete sycotic cause; that introduced into the human organism it had the power to produce all the fearful train of diseases expressed by the term *Sycosis;* that the

*wart* is the external specific representative of the internal sycotic condition. It will be further remembered that this child was perfectly healthy, even more than commonly strong and robust, up to the time of its vaccination; then began the long train of evils which caused her so much of suffering and her parents anxiety and her doctor of study and perplexity; that when her recurring attacks were apparently cured, the *child* was only partially cured by remedies only like a part of her sick condition, one most essential part being omitted in gathering the symptoms, and, of course, in selection of the remedies employed in treating these paroxysms; that as a result of this omission the unrecognized element progressed in its invasion of the organism, making deeper inroads upon it till destruction so important as that of the hip-joint was threatened, which had already become much diseased. The sycotic cause and condition were singularly overlooked by Dr. Dunham and myself, and it was only when this was apprehended that the true remedy was found and the cure was made promptly and perfectly. It appears, on looking at the history of the case and its partially successful treatment before this condition was apprehended, that if this had not happened the joint would have been destroyed, if not even the life of the child, after great and long suffering.

The above is a true picture of a case taken from life. The existence, importance, potency, and origin of this third chronic miasm could hardly be more clearly demonstrated than it was in this case; its origin from vaccination (*vide* Bœnninghausen and Wolf); its potency in the often inveterate and always troublesome eczema, in the croup, laryngismus stridulus, and the disease of the hip-joint; its importance in the sufferings and threatened life of the little patient. We say this case demonstrates these facts and also the powers of the truly antisycotic remedy, when found and administered in accord with the requirements of homœopathic law. If one is disposed still to deny the antisycotic element in the ultimate remedy prescribed, and to say the partial results which followed prescriptions in the croup and spasmodic attacks were owing to careless prescribing, and that the cure would have been effected in the beginning if the truly homœopathic remedy had been given irrespective of the sycotic element of the case, let him remember Dunham did his best, without considering this element, and Dunham was neither a weak man nor a careless prescriber; let such an objector show a better or a more careful, and then find all the fault with him and the other prescribers for the case he feels compelled to.

The result of the last prescription demonstrates the verity of the miasm, sycosis, and the power of the antisycotic remedy. It will not, I think, be doubted by any candid and intelligent homœopathist, in view of the partial results of prescriptions from one so truly a master in prescribing as Dunham, that, wanting the antisycotic given at last, the case would have terminated fatally. This, when given, wrought a prompt and perfect cure. In view of such evidence as this case presents, is it not pitiful that there are those who claim to be recognized as representatives of homœopathic philosophy and practice, and yet talk of Hahnemann's chronic miasms as "errors," "fancies," and "fallacies," and publicly boast they are not "weighted down," by these or kindred elements of our philosophy, but seem to rejoice in such freedom as ignorance and conceit can give them? They even affect to look down on the venerable old master and the glories of his discoveries as matters far beneath their standpoint of professional philosophy—these men, whose only professional importance is derived from a *name* they have misappropriated from him. Is it not pitiful?

